# Interpretation of intraoperative parathyroid hormone monitoring according to the Rome criterion in primary hyperparathyroidism

**DOI:** 10.1038/s41598-022-07380-4

**Published:** 2022-02-28

**Authors:** Giuseppa Graceffa, Calogero Cipolla, Silvia Calagna, Silvia Contino, Giuseppina Melfa, Giuseppina Orlando, Riccardo Antonini, Alessandro Corigliano, Maria Pia Proclamà, Sergio Mazzola, Gianfranco Cocorullo, Gregorio Scerrino

**Affiliations:** 1 Department of Surgical Oncology and Oral Sciences, Unit of Oncological Surgery, University of Palermo, Via L. Giuffré, 5, 90127 Palermo, Italy; 2 Department of Surgical Oncological and Oral Sciences, Unit of General and Emergency Surgery, Policlinico “P. Giaccone”, University of Palermo, Via Liborio Giuffré 5, 90127 Palermo, Italy; 3 Unit of Clinical Epidemiology and Tumor Registry, Department of Laboratory Diagnostics, Policlinico “P. Giaccone”, University of Palermo, Via L. Giuffré, 5, 90127 Palermo, Italy; 4 Department of Surgical Oncological and Oral Sciences, Unit of Endocrine Surgery, University of Palermo, Via L. Giuffré, 5, 90127 Palermo, Italy

**Keywords:** Biomarkers, Endocrinology

## Abstract

Intraoperative parathyroid hormone dosage allows real-time monitoring of the decrease in PTH levels during parathyroidectomy and verify procedure’s efficacy. Currently, none of the interpretative criteria used has absolute accuracy. The aim of this study is to evaluate diagnostic accuracy of the Rome criterion verifying diagnostic significance of the individual assays. A total of 205 patients with primary hyperparathyroidism from a single adenoma were retrospectively evaluated and monitored with baseline PTH, PTH at 10 min and PTH at 20 min after adenoma excision. The accuracy of the latter two assays compared with baseline was compared by ROC curves. In addition, was evaluated the influence on these data of localization diagnostics (ultrasounds and scintigraphy), definitive histology, and type of surgery performed. The ratio of 20-min sampling to baseline in the Rome criterion showed highest diagnostic significance. This finding was not influenced by the type of surgery performed, definitive histologic examination, or intraoperative localization of the adenoma. The Rome criterion has shown its high reliability in detecting persistence. The ratio of sampling at 20 min to baseline is by far the best performing. Further studies are needed to evaluate whether sampling at 10 min after adenoma excision can be considered not mandatory.

## Introduction

The need of parathyroid hormone (PTH) value measurement during parathyroidectomy has been highlighted since the late 1980s^1,2^. In fact, although parathyroidectomy (PTx) is successfully in 90% of cases of primary hyperparathyroidism (HPT1), in 10% the calcemia and PTH values return or become elevated as a result of an unidentified or inaccessible adenoma, multiglandular disease, insufficient excision of hyperplastic glands, difficulty in distinguishing between adenomatous tissue and hyperplastic gland^3^. In 1993, Irvin et al. developed the rapid intraoperative PTH assay^4,5^. The Quick intact intraoperative parathormone assay (ioPTH) is a technique that allows real-time monitoring of the decline in plasma PTH levels as the glands are removed during PTx^6,7^. At present, a PTH threshold that can predict 100% of cases of a persistent/recurrent disease is not well defined^8,9^. Therefore, several interpretative models of the ioPTH curve have been proposed^10^. Among these, at our institution we have been adopting for more than a decade the Rome criterion, which is based on a drop > 50% of ioPTH values compared to the highest pre-excision values and/or ioPTH value within the reference threshold, and/or ≤ 7.5 ng/L lower than the value at 10 min post-excision, 20 min after excision^11,12^.

The introduction of ioPTH and the optimization of its interpretative models, together with the refinement of techniques for localization of pathological parathyroid glands have allowed the implementation of focused approach for PTx^13,14^ and the extension of surgical indications, with an overall improvement of treatment optimization, especially in terms of complications of HPT1^15^. In addition, ioPTH may be a useful tool in difficult settings, such as concurrent thyroid disease or unlocalized parathyroid gland^16,17^.

Overall, the routine use of ioPTH is justified by the usefulness shown in decision making in HPT1 surgery^18^.

Despite the effectiveness of ioPTH in optimizing results in HPT1 surgery, this practice results in increased costs, given by the combination of the cost of instruments, consumables, and the time to devote to each analysis^19^. The last two items are directly proportional to the number of samples analysed in each procedure.

The aim of our study is to verify, in the context of a procedure monitored according to the Rome criterion, which requires at least three intraoperative samplings, which one correlates most closely with the cure of HPT1, and whether this result alone can be judged sufficiently accurate to make the other unnecessary.

## Methods

### Study population

The Rome criterion has been used almost systematically since 2010 at all Surgical Units of our Department; however, we excluded from the study patients treated in the 3-year period 2010–2012 because we considered this period necessary for the development and internal validation of the protocol.

The prospectively collected medical records of a retrospective cohort of patients undergoing PTx from 2013 to 2020 at the three Surgical Units of the Department of Surgical, Oncological and Oral Sciences of the Policlinico Universitario “P. Giaccone”of Palermo involved in the treatment of endocrine diseases was evaluated in this study. Our tertiary hospital is a referral center for the treatment of endocrine disorders. The volume of parathyroidectomies performed in the last decade at our department, can be defined as intermediate, although it collects the largest proportion of PTx performed in western Sicily^20–22^.

We excluded from the study patients with secondary or tertiary hyperparathyroidism, redo- surgery, Multiple Endocrine Neoplasia, patients with incomplete records, missing data and/or ioPTH monitoring not in agreement with standards of Rome criterion.

We also excluded cases with double adenoma, which we considered a confounding factor because the timing of the samples could not be interpreted according to the Rome criterion.

In all patients undergoing surgery, vitamin D values were previously corrected to within the normal range (30–100 ng/ml). Patients with vitamin D values below the normal range were not evaluated for the study.

### Surgical techniques

The patients included in this study underwent three different surgical techniques: conventional bilateral exploration, minimally invasive video-assisted parathyroidectomy (both under general anesthesia), focused open minimally invasive parathyroidectomy (under local anesthesia).

Bilateral conventional exploration was preferred in cases of doubtful or absent localization, or in most cases where an intrathyroid site of the parathyroid was suspected. This technique was also employed in the presence of mediastinal scintigraphic uptake. It was performed with a 3.5–4 cm Kocher incision, preparation of flap below the platysma, and access to the thyroid beds bilaterally after separation of the strap muscles on the midline. MIVAP was preferred in the presence of a parathyroid with probable localization (clear US image or scintigraphy uptake), or certain (the two imaging techniques concordant), in this case if the parathyroid was a superior one (PIV), given its location deep in the neck and anterior to the recurrent nerve. In addition, this technique was preferred in cases of patients not compliant with local anesthesia. Some patients with intrathyroid parathyroid, suspected at US and confirmed at scintigraphy, underwent video-assisted hemithyroidectomy necessary to remove enlarged parathyroid gland.

The focused technique under local anesthesia was chosen in the presence of concordance of imaging techniques, for lower, superficial, non-mediastinal parathyroid glands, in consenting and compliant patients.

### Intraoperative parathyroid hormone monitoring

The total intact PTH assay was performed with immunoradiometric assay (IRMA). The blood samples were processed at the hospital's centralized laboratory. This allowed for the best possible standardization on results across the three clinical institutions involved in patient recruitment.

Regardless of the surgical technique used, a PTH drop considered insufficient in both 10' and 20' sampling led to a continuation of the dissection and, in some cases, the conversion from a focused or minimally invasive technique to a conventional technique, generally aimed at bilateral exploration.

### Data collection

**Figure 1:**
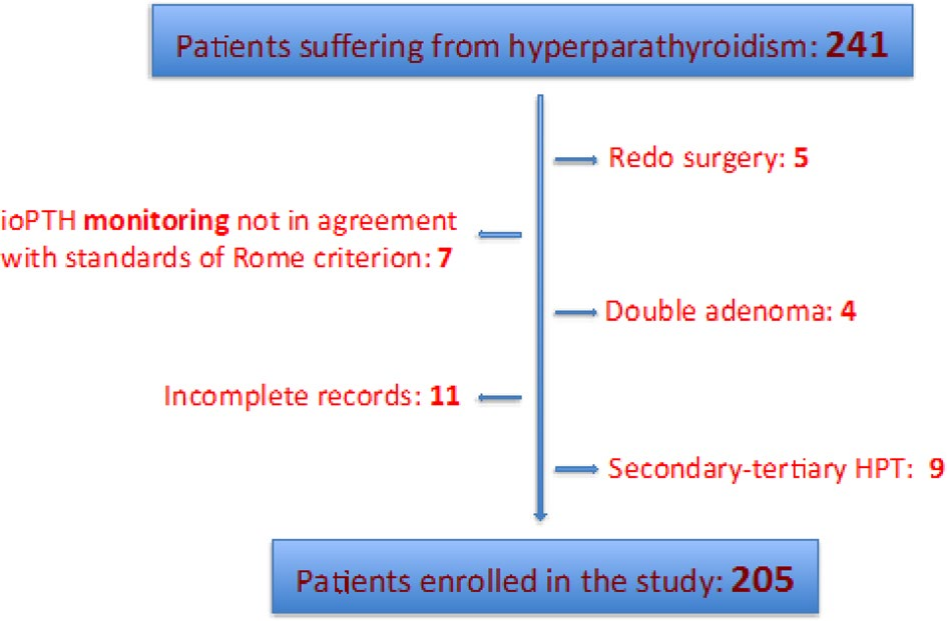
Enrollment process and causes of exclusion.

The clinical records of 241 patients suffering from hyperparathyroidism, enrolled for surgery at the Department of our Department from 2013 to 2020 were examined. We excluded 9 patients with secondary or tertiary hyperparathyroidism, 7 patients because the ioPTH monitoring was not in agreement with standards of Rome criterion, 5 patients undergoing reoperation, who had undergone the first surgical procedure in another hospital, 4 patients with double parathyroid adenoma, 11 patients who presented incomplete records. After case selection according to the inclusion/exclusion criteria, a total of 205 patients were included in the study (Fig. 1).

Demographics (age, gender, and year of operation) were taken into account, as well as baseline PTH (range: 15–65 pg/ml), PTH values 10 min after PTx, and PTH values 20 min after PTx. We also recorded the surgical technique adopted, the results of imaging (US, scintigraphy), and the concordance of results between the two imaging techniques, as well as the definitive histological diagnosis.

Surgical treatment outcome was recorded as a dichotomous variable (cure/persistence). We considered persistence as the increase of PTH levels above the upper normal limit (> 65 pg/mL) with any calcemia values (normal range: 8.6–10.0 mg/dL) and vitamin D values above the threshold of sufficiency (> 30 ng/ml). PTH and calcemia were evaluated on the first postoperative day and 30 days after surgery. In the latter sample, vitamin D was also evaluated.

The variables were recorded on an excel spreadsheet.

### Outcomes

The primary outcome of the study was the significance of the result of each postoperative PTH assay, compared with baseline sampling, in predicting cure or persistence.

We also evaluated whether the surgical technique adopted, the histology, or the localization imaging results could in any way influence the outcome.

### Statistical analysis

In a first step, a univariate analysis was performed. Fisher's exact test for categorical variables and the Mann–Whitney test for continuous variables were applied.

Next, ROC curves were generated in order to evaluate the accuracy of changes in PTH (T10 minutes postoperative) or PTH (T20 minutes postoperative) relative to baseline PTH as a predictor of the occurrence of disease persistence. These percentage changes were derived through the following formulas:

$$ \begin{aligned} & {\text{PTHT10-baseline PTH Ratio}} = \, \left( {{\text{PTHT10-baseline PTH}}} \right)/\left( {\text{baseline PTH}} \right) \, \times {1}00 \\ & {\text{PTHT20-baseline PTH Ratio}} = \, \left( {{\text{PTHT20-baseline PTH}}} \right)/\left( {\text{baseline PTH}} \right) \, \times 100 \\ & {\text{PTHT20-PTHT1}}0{\text{ Ratio}} = \, \left( {{\text{PTHT20-PTHT1}}0} \right)/\left( {\text{baseline PTH}} \right) \, \times 100 \\ \end{aligned} $$

A multivariate logistic analysis was performed in which the following variables were included: Age, Sex, PTHT10-baseline PTH Ratio, PTHT20-baseline PTH Ratio, PTHT20-PTHT10 Ratio, US, Scintigraphy, US + scintigraphy, and Localization.

In addition, the Mann–Whitney test was applied to evaluate a possible relationship between the continuous variable PTHT20-baseline PTH Ratio with the result of scintigraphy, the only variable to be significant in both univariate and multivariate analysis, to assess their possible interrelation.

Statistical analyses were performed with RStudio software (version 3.4.1 of 2017–06-30) for R (version 2.1) (25, 26). The application software "pROC" was used to generate ROC curves.

The present study involved uman participants and was conducted in conformity with ethical and human principles of research according to the ethical standards of the Declaration of Helsinki of 1964 and its amendments.

Informed consent was obtained from all individual participants included in the study according to format of "Società Italiana di Endocrinochirurgia" (SIEC), "Club delle Unità di Endocrinochirurgia" (UEC) and, from 2017, "Società Italiana Unitaria di Endocrinochirurgia" (SIUEC).

Ethical Board approval was obtained by "Comitato etico del Policlinico Paolo Giaccone—Palermo".

## Results

From the univariate analysis, the variables that seem to be risk factors for disease persistence are: sex (males seem to be more at risk compared to females), PTH-T10, PTH-T20, US (negative), Scintigraphy (negative), US + Scintigraphy (not in accordance) and localization (intrathyroid parathyroid gland) (Table 1).

**Table 1:** On univariate analysis, both averages of the 10 min' and 20 min' detections were significant.

Variable	Cure	Persistence	Total	OR (IC95%)	*p* value
**Univariate analysis**					
Age (mean)	57	58			**0.6**
**Sex**					
M	38	6	44	3.45 (0.90–12.77)	0.03623
F	154	7	161		
Total	192	13	205		
Baseline PTH	183.3	189.3			0.8054
PTH T10’	62.65	91.3			**0.00059**
PTH T20’	29.34	91.0			**8.518e−07** **< 0.0001**
**Technique**					
Bilateral exploration	139	12	151		0.2839
MIVAP	28	0	28		
Focused	25	1	26		
Total	192	13	205		
**Histology**					
1	37	5	42		0.4188
2	136	8	144		
3	11	0	11		
4	8	0	8		
Total	192	13	205		
**US**					
Localized	184	1	185	**249.8 (31.7–10,788.6)**	**1.901e−13** **< 0.001**
Not localized	8	12	20		
Total	192	13	205		
**Scintigraphy**					
Localized	157	4	161	**9.93 (2.59–46.7)**	**0.0001701**
Not localized	35	9	44		
Total	192	13	205		
**US + scintigraphy**					
Confirmed	154	1	155	47.5 (6.7–2067.4)	**1.559e−07** **< 0.0001**
Not confirmed	38	12	50		
Total	192	13	205		
**Localization**					
Cervical	171	5	176		**7.829e−05** **< 0.0001**
Intrathyroid	17	7	24		
Mediastinum	4	1	5		
Total	192	13	205		

Equally significant was the difference between the number of patients in whom localization diagnostics (ultrasounds, scintigraphy) were effective, compared with cases with no localization. Patients with ectopic, intrathyroidal or mediastinal localizations had a significantly higher rate of persistence; finally, this rate was not significantly different according to histology.

*MIVAP* minimally invasive video assisted parathyroidectomy, *Histology* *1* Hyperplasia, *2* adenoma, *3* atypical adenoma, *4* carcinoma; *US* ultrasounds.

Significant values are in bold.

**Figure 2:**
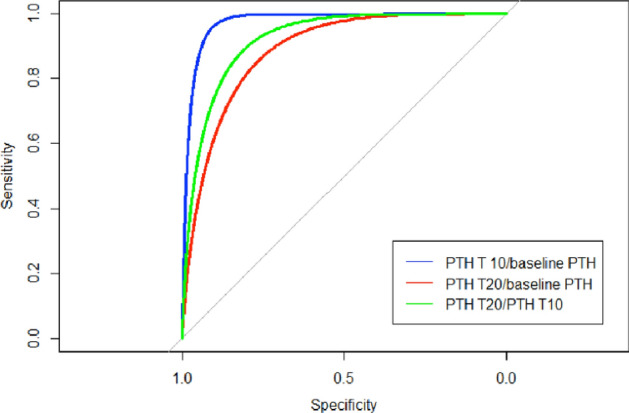
Calculation with ROC curves of the relationship between sensitivity and specificity of the PTH T10/baseline PTH, PTH T20/PTH T10, and PTH T20/baseline PTH ratios. The latter curve includes the area below with the greatest extension, demonstrating the greater diagnostic significance of this detection compared with the others, although these also showed excellent performance.

When comparing the three ROC Curves, we see that the decrease rate in PTH-T10 versus baseline PTH appears to be a moderately accurate predictor of disease persistence with AUC = 0.87 and 95% CI (0.79–0.95) with a cut-off value of -58,4%, Specificity = 0.66 95% CI (0.59–0.73) and Sensitivity = 1 with 95% CI (1–1); whereas the decrease rate in PTH-T20 compared to baseline PTH appears to be a highly accurate predictor of disease persistence with AUC = 0. 98 and 95% CI (0.92–0.99) with a cut-off value of − 63.5%, Specificity = 0.95 95% CI (0.92–0.98) and Sensitivity = 0.92 with 95% CI (0.77–1) (Fig. 2).

Finally, the decrease rate in PTH-T20 versus PTH-T10 (PTH ratio) turns out to be a highly accurate predictor of disease persistence with AUC = 0. 92 and 95% CI (0.84–0.97) with a cut-off value of − 35.5%, Specificity = 0.79, 95% CI (0.73–0.89) and Sensitivity = 0.92 with a 95% CI (0.77–1).

In multivariate logistic analysis, the following variables were included: Age, Sex, Baseline_PTH-T20_PTH ratio, Scintigraphy and Localization. The model revealed that the decrease rate PTH-T20—baseline PTH and scintigraphy are risk factors for disease persistence with OR and 95%IC of 1.124 95%IC (1.067–1.219) (*p* value < 0.05) and 30.87 95%IC (2.684–1.05e+03) (*p* value < 0.05), respectively (Table 2).

**Table 2:** Also on multivariate analysis, high significance was observed for both PTH T20/baseline PTH ratio and scintigraphy (higher risk of persistence in the absence of localization on scintigraphy).

Variable	OR	CI (Inf) 95%	CI (Sup) 95%	*p* value
**Multivariate analysis**				
Age	0.884	0.759	0.988	0.057494
Sex	1.823	0.138	20.16	0.625675
PTH T20/baseline PTH ratio	1.124	1.067	1.219	0.000396*
Scintigraphy not localized	30.87	2.684	1.05e^+0.3^	0.016941*
Intrathyroid localization	9.526	0.960	1.11e^+0.2^	0.051673
Mediastinal localization	14.82	0.676	3.47e^+0.3^	0.400633

Both of these parameters were marked (*).

**Figure 3:**
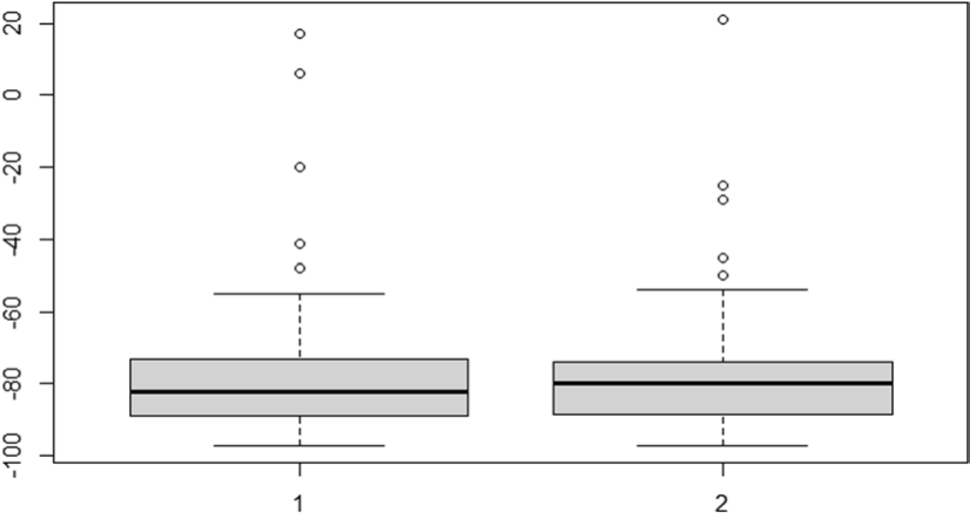
Grouping in boxplots concerning the distribution of Ratio_PTHT20/baseline PTH according to the scintigraphy outcome (1 = localized gland at scintigraphy; 2 = non-localized gland at scintigraphy). It is evident that concentrations, median and third quartile in the two boxes are extremely similar.

No correlation between categorical variables and the PTHT20-baseline PTH Ratio was shown. In particular, considering it reasonable to hypothesize a possible correlation between the outcome of scintigraphy (theoretically correlated in a directly proportional relationship to the metabolic activity of the parathyroid adenoma) and the extent of PTH decrease, we report in detail the relevant statistical evaluations. The groups of subjects with diagnostic scintigraphy (group 1) and those with non-diagnostic scintigraphy (group 2) were similar in terms of ratio (Fig. 3).

The indicators, for the two groups, were:

- *Group 1* M = − 79.38%; Median = − 82.00%; 1st Quartile = − 89.00%; 3rd Quartile = − 73.00%.
- *Group 2* M = − 74.93%; Median = − 80.00%; 1st Quartile = − 88.25%; 3rd Quartile = − 75.00%.

## Discussion

Parathyroid surgery is considered a procedure whose strategic approach requires considerable specific expertise, especially in choosing the indication for surgery and the technique to use, as well as determining the extent of dissection^3,8,22,23^. The development of localization imaging has unquestionably improved the chances of correctly identifying hyperfunctioning parathyroid tissue^2,3,13,23,24^. IoPTH has attracted interest since its launch because of its capacity to validate, during the execution of surgery, the removal of all pathological parathyroid tissue or the incompleteness of the procedure^4,5^. In fact, the short half-life of PTH allows us to assess its kinetics in just a few minutes^1,2,25^. Standardized criteria for ioPTH monitoring have been proposed, but none of them has absolute diagnostic accuracy^10–12^. The criteria commonly suggested for the interpretation and application of ioPTH kinetics in clinical practice are essentially based on four cornerstones: the decrease rate from the maximum value achieved starting from the induction of anesthesia; the time span between samplings; the range of normality (whose maximum value is usually considered 65 pg/mL); and the absolute post-excision value considered safe. The first and second groups include the criteria of Miami (ioPTH drop ≥ 50% from any highest pre-excision level at 10 min after gland excision)^26^, Vienna (ioPTH drop ≥ 50% within 10 min following gland removal)^27^, and Rome (ioPTH drop > 50% from the highest pre-excision level, and/or ioPTH within the range of 20 min after gland removal, and/or ≤ 7.5 ng/L lower than the 10 min post removal value). The latter criterion has enhanced, among the rules, the achievement of the maximum threshold value of normality^11^. Finally, a recent study demonstrated the extremely low likelihood of disease persistence in patients in whom an ioPTH value of 40 pg/mL was reached within 15 min^18^.

We chose the Rome criterion rather than others because of the minimization of false positives, which protects against possible persistence of residual pathological tissue (i.e.: "sleeping double adenoma")^10,11^. The Rome criterion appears to provide the best success rate in avoiding persistence, although it could result in extended operating times and a greater number of intraoperative samplings required and, consequently, higher costs^28^. In fact, the authors who proposed this criterion showed that the predictive factors of disease persistence are a drop in ioPTH values that does not reach 50% after 20 min or an ioPTH value that remains, in the same time frame, above the maximum threshold (65 pg/mL). A third criterion introduced is the comparison of ioPTH values at 10 min, which could be significantly higher than at 20 min in the case of persistence^11^.

Several factors must be considered in support of extended monitoring: clinical conditions, such as renal failure, vitamin D deficiency, diabetes, and obesity, may require a prolongation of monitoring with an increase in the number of samples, either by modifying the kinetics of PTH or for other causes^29–32^.

Our results confirm the importance of extending the sampling up to 20 min and the significance of the ratio ioPTH T20-ioPTH T10. However, the latter does not appear to produce any additional accuracy compared to the ratio of ioPTH T20-baseline PTH. From these results, it that ioPTH is a good indicator to assess the recovery or possible persistence of the disease. However, among the various indicators, the most reliable appears to be the variation in PTH value detected at 20 min after surgery compared to baseline PTH. The detection of PTH at 10 min, according to our data, would lose its importance.

It could be noted that the prolongation of ioPTH monitoring to 20 min leads to an increase in costs. It has been estimated that the greatest impact on costs depends on the number of samples taken during an intervention rather than the time spent for monitoring^33^.

We emphasize that there is no interrelationship between the ratio ioPTH T20-baseline PTH and false negatives of localization imaging (scintigraphy). We found this finding to be interesting, because one might consider that increased parathyroid tissue activity, to which a more intense uptake might correspond, does not appear to be related to PTH kinetics. It also appears clear that the higher persistence rate with ectopic pathological parathyroid localizations (intrathyroid, mediastinal) refers to sporadic cases with a higher failure rate, which is consistent with literature data^34–36^.

This study has some limitations: the limited number of patients enrolled in the study; the exclusion of the cases of double adenoma, considered a confounding factor but which, nevertheless, constitutes the main context at risk of persistence more than all the others; and the relatively high number of procedures performed with bilateral exploration, prevalent especially in the early years of the study, compared with minimally invasive techniques or focused procedures. This is not, at present, the standard in our institution as well as in centers with medium or high volumes of activity. Finally, no distinction was made between the different baseline values of PTH, varying from 80 to about 1000 pg/ml: in this regard, we believe that such a wide variability includes subgroups that could express different kinetics of PTH.

However, the value of our data lies in their origin in a real scenario, in which the volume of activity is to be considered well above the threshold of low-volume centers, but not fully classifiable in high-volume centers, whose number, at least in Italy, is very limited and the minimum threshold considered is not achievable, at least at this time, in some areas of the country, such as Sicily.

## Conclusion

The data emerging from our study in favor of extending ioPTH monitoring to 20 min after baseline detection appeared to be quite robust. Closer evaluation revealed the highest diagnostic significance of the later detection compared with the others.

However, the abolition of the 10-min sampling is not part of our routine, it is only a statistical evaluation carried out on our data, evaluated in any case according to the criterion of Rome, which at the moment, at our institution, continues to be the preferred for ioPTH monitoring.

However, we believe that an evaluation of a larger number of patients, possibly recruited for a multicentre study, could allow the validation of a simpler method in the evaluation of ioPTH monitoring with a small but significant cost reduction.
